# Prevalence and factors associated with modern contraceptives utilization among female adolescents in Uganda

**DOI:** 10.1186/s12905-021-01206-7

**Published:** 2021-02-10

**Authors:** Quraish Sserwanja, Milton W. Musaba, David Mukunya

**Affiliations:** 1 Monitoring and Evaluation Department, Doctors With Africa, CUAMM, TM Lion Hotel, Juba, South Sudan; 2 Department of Obstetrics and Gynaecology, Busitema University, Tororo, Uganda; 3 Department of Public Health, Busitema University, Tororo, Uganda; 4 Sanyu Africa Research Institute, Mbale, Uganda

**Keywords:** Adolescents, Contraceptives, Utilization, Uganda

## Abstract

**Background:**

The sexual and reproductive health (SRH) needs of adolescents remain largely unmet. For instance, over 20 million female adolescents in need of, a modern contraceptive method are not using any. This study determined the factors associated with utilization of modern contraceptives among female adolescents in Uganda.

**Methods:**

A cross sectional study was conducted using the Uganda Demographic and Health Survey (UDHS) 2016 data of 4, 264 adolescents aged 15 to 19 years. Multistage stratified sampling was used to select study participants. Multivariable logistic regression was used to determine the factors associated with modern contraceptive utilization. All our analyses were done using SPSS version 25.

**Results:**

The prevalence of modern contraceptive utilization among female adolescents was 9.4% (401/4264: (95% CI: 8.6–10.3). The odds of contraceptive utilisation were 1.6 times (AOR = 1.60; 95% CI: 1.09–2.34) higher among married adolescents compared to unmarried adolescents. Adolescents whose age at first birth was less than 15 years (AOR = 2.01; 95% CI: 1.01–3.99) were twice more likely to utilize a modern contraceptive compared to those whose age at first birth was above 15 years. Women belonging to the Central region (AOR = 1.93; 95% CI: 1.01–3.69) and those in the middle wealth quintile (AOR = 1.91; 95% CI: 1.06–3.46) were 93% and 91% more likely to utilize a modern contraceptive compared to those in the Northern region and those in the poorest wealth index respectively.

**Conclusion:**

The prevalence of modern contraceptive utilization was 9.4%. The findings show the need for designing targeted interventions due to differences in adolescents according to their wealth index, regions and marital status.

## Background

Adolescence is a crucial period during which many begin sexual activity [1, 2]. Globally, the sexual and reproductive health (SRH) needs of adolescents have remained largely unmet with about 20 million female adolescents aged 15–19 years in need of modern contraceptive methods [3]. Sub-Saharan Africa has the highest rates of adolescent pregnancy and the lowest utilization of modern contraceptives [1, 2, 4]. A third of adolescent pregnancies in Sub-Saharan Africa are unintended, with over a third of these unintended pregnancies being unwanted and end up as unsafe terminations [1, 4]. Pregnancy and childbirth related complications are the leading causes of maternal death among female adolescents aged 15–19 in low and middle-income countries (LMICs) [5, 6]. Adolescent pregnancy and childbirth is associated with other poor health outcomes such as anemia, preterm birth, low birthweight, adverse adolescent mental health effects, besides its negative effects on higher education attainment and job opportunities [1, 2, 7–10].

Use of modern contraceptives reduces maternal mortality, improves health outcomes of adolescent mothers and their children and reduces the costs associated with teenage pregnancy [5, 11]. Planned childbirths increase the likelihood of attaining higher educational levels, which results in financial independence [12–14]. Despite the global progress in increasing availability and coverage of family planning services, [5, 15] most of the contraceptive needs of adolescents are largely unmet [1, 5, 11]. Uganda’s adolescent fertility rates are among the highest in the world [13, 16, 17]. According to the latest Uganda Demographic and Health Survey (DHS), 24.8% of girls aged 15–19 had already begun childbearing [18]. Furthermore, Uganda has one of the lowest contraceptive prevalence rate in the region [16]. The high numbers of adolescents giving birth at an early age partly contributes to Uganda’s high fertility levels [19].

The Government of Uganda through the ministry of Health and its development partners have come up with different initiatives to ensure improved distribution, access and utilization of contraceptives, however, the contraceptive prevalence rate remains low while the unmet need is high [13, 20]. Studies in Uganda have focused mainly on knowledge, attitudes and barriers to access among older women and others have been predominantly qualitative in nature [13]. For Uganda’s health system to effectively respond to the high and increasing population of adolescents, its crucial to understand the predictors of modern contraceptive utilization among female adolescents. This study aimed at describing factors associated with utilization of modern contraceptives among female adolescents aged 15–19 years. Its recommendations will help in improving implementation of programs focusing on the reproductive health needs of female adolescents in Uganda, which with better health, this will increase their contribution to the development of the country.

## Material and methods

### Study design

We conducted a secondary data analysis of the 2016 Uganda demographic health survey (UDHS) data set.

### Study data

We used the 2016 Uganda Demographic and Health Survey (UDHS) dataset for this study after obtaining permission from the MEASURE DHS project. MEASURE DHS program conducts periodical nationally representative cross-sectional household surveys in low and middle income countries (LMICs) standardized to enable comparisons among the different countries [21]. The surveys are usually conducted after every five years [21].

The UDHS data were collected from June to December 2016 [22]. UDHS was implemented by the Uganda Bureau of Statistics (UBOS) with the technical assistance of Inner City Fund (ICF) International through the USAID-supported MEASURE DHS project [22]. The UDHS inquired about household members’ and individuals’ socio-demographic and reproductive health information using household, women’s, men’s and biomarker questionnaires [22, 23]. The data used in this study was collected using the women’s questionnaire. Female adolescents were asked if they were currently using any method of modern contraception [22].

### Study sampling and participants

The UDHS used two-stage systematic sampling to select participants from households nested in clusters (enumeration areas) across the all the regions of Uganda [22, 23]. The 2014 population and housing census sample frame was used to select the enumeration areas [22]. UDHS 2016 included women aged 15 to 49 years who were either permanent residents or slept in the selected household the night before the survey [22]. In this study, we included female adolescents aged 15–19 years who responded to the women’s questionnaire. The UDHS interviewed 18,506 women aged 15–49 years after administering informed consent [22]. Of the 18,506 women, 4,264 were female adolescents aged between 15 and 19 years [22].

### Outcome variables

Utilization of any method of modern contraceptive method was coded as one (1) while non-utilization was coded as zero (0).

### Explanatory variables

This study included determinants of modern contraceptives utilization basing on evidence from available literature and data [3, 13, 19, 22]. Nine explanatory variables were included in the analysis as shown in Additional file 1: Table 1. Wealth index is a measure of relative household economic status and was calculated by DHS from information on household asset ownership using Principal Component [22].

**Table 1:** Background characteristics of Ugandan female adolescents as per the 2016 UDHS

Characteristics		N = 4264		%
**Age**				
15 to 17		2629		61.6
18 to 19		1636		38.4
**Residence**				
Urban		1034		24.3
Rural		3230		75.7
**Region**				
Western		982		23.0
Eastern		1248		29.3
Central		1132		26.6
Northern		902		21.2
**Age at First Birth**^**a**^				
Less than 15		53		01.2
15–19		773		18.1
**FP Counselling in the year**				
Yes		514		12.1
No		3750		87.9
**Marital status**				
Married		850		19.9
Not Married		3414		80.1
**Working**				
Yes	2059		48.3	
No	2205		51.7	
**Education level**				
No Education		76		01.8
Primary Education		2759		64.7
Secondary Education		1351		31.7
Higher		78		01.8
**Wealth Index**				
Poorest		764		17.9
Poorer		840		19.7
Middle		815		19.1
Richer		854		20.0
Richest		990		23.2
**Modern contraception use**				
Yes		401		09.4
No		3863		90.6

^**a**^Age at birth not applicable to 3438

### Statistical analysis

To ensure validity of our study findings, sampling weights provided by UDHS were used. Use of sample weights helped to account for the unequal probability sampling in different strata [24] and to ensure representativeness of the survey results at the national and regional level [25]. We used Complex sample analysis was performed using SPSS version 25.0 statistical software to account for the multi-stage cluster study design. Proportions were tabulated for each of the categorical independent variable. Each exposure was assessed separately for its association with the outcome variable using bivariable logistic regression and we presented crude odds ratio (COR), 95% confidence interval (CI) and p-values. Independent variables found significant at bivariable level (p-value < 0.25) and those found significant in similar context studies were included in the final multivariable logistic regression model. Adjusted odds ratios (AOR), 95% Confidence Intervals (CI) and p-values were calculated with statistical significance level set at *p* value < 0.05. All variables in the model were assessed for collinearity, which was considered present if the variables had a variance inflation factor (VIF) greater than 10.

## Results

### Sociodemographic characteristics of Study Participants

A total of 4,264 women were included in this study **(**Table 1). Of these, 401 (9.4%) (95% CI: 8.6–10.3) were utilizing a modern contraceptive. Majority of the adolescents were residing in rural areas (75.7%), aged 15–17 years (61.6%), not married (80.1%), had seven or less years (primary) of education (64.7%) and belonged to the richest wealth quintile (23.2%).

### Factors associated with modern contraceptive utilization

Factors associated with modern contraceptive utilization were: marital status, age at first birth, region and wealth index as indicated in Table 2.

**Table 2:** Predictors of modern contraception use among female Ugandan adolescents

Characteristics	Crude model (n = 4264) COR (95% CI)	*P* value	Adjusted odds ratio (n = 4264) AOR (95% CI)	*P* value
**Age**				
15 to 17	1	< 0.001	1	0.468
18 to 19	**4.18 (3.18–5.49)**		1.19 (0.74–1.90)	
**Education level**				
No education	1	0.008	1	0.386
Primary	2.14 (0.74–6.18)		1.75 (0.55–5.53)	
Secondary	2.97 (0.99–2.19)		2.45 (0.72–8.32)	
Higher	**5.58 (1.57–19.86)**		2.25(0.16–2.23)	
**Marital Status**				0.016
No	1	< 0.001	1	
Yes	**3.69 (2.84–4.79)**		**1.60 (1.09–2.34)**	
**Region**				
Northern	1	0.001	1	0.386
Eastern	**1.69 (1.18–2.43)**		0.97 (0.55–1.72)	
Western	1.22 (0.79–1.89)		1.16 (0.62–2.18)	
Central	**2.19 (1.46–3.28)**		**1.93 (1.01–3.69)**	
**FP counselling**				
No	1	< 0.001	1	0.350
Yes	**3.04 (2.24–4.12**		1.19 (0.82–1.73)	
**Wealth Index**				0.242
Poorest	1	0.308	1	
Poorer	1.36 (0.89–2.06)		1.57 (0.89–2.76)	
Middle	1.48 (0.99–8.87)		**1.91 (1.06–3.46)**	
Richer	1.46 (0.96–2.23)		1.36 (0.71–2.63)	
Richest	1.54 (0.98–2.39)		1.85 (0.89–3.84)	
**Working**				
No	1	< 0.001	1	0.352
Yes	**2.02 (1.56–2.62)**		1.21 (0.81–1.81)	
**Age at first birth**		0.083		0.046
15–19	1		1	
Less than 15	**1.73 (0.93–3.19)**		**2.01 (1.01–3.99)**	

bold = Significant

Married adolescents (AOR = 1.60; 95% CI: 1.09–2.34) were 60% more likely to utilize a modern contraceptive compared to non-married adolescents. Adolescents whose age at first birth was less than 15 years (AOR = 2.01; 95% CI: 1.01–3.99) were twice as likely to utilize a modern contraceptive compared to those whose age at first birth between was 15–19 years. Women belonging to the Central region (AOR = 1.93; 95% CI: 1.01–3.69) were 93% more likely to utilize a modern contraceptive compared to those in the Northern region. Women belonging to the middle wealth quintile (AOR = 1.91; 95% CI: 1.06–3.46) were 91% more likely to utilize a modern contraceptive compared to those in the poorest wealth index.

## Discussion

This study investigated the prevalence and factors associated with modern contraceptive utilization among Ugandan female adolescents aged 15–19 years. Prevalence of modern contraceptive utilization was 9.4% (95% CI: 8.6–10.3). This is very low and negatively affects Uganda’s progress of achieving sustainable development goal (SDG) 3 target 3.7 aimed at ensuring universal access to sexual and reproductive health-care services, including for family planning by 2030 [26]. With low contraceptive utilization, and with Uganda having some of the highest adolescent fertility rates in the region, these findings imply that a lot has to be done to ensure increased access and utilization of contraceptives as one of the ways of reducing teenage pregnancies and the associated morbidity and mortality if we are to meet the SDG targets as a country.

Our study in comparison to other studies showed that contraceptive utilization in Uganda is lower than that of Kenya, Rwanda, Tanzania, Europe, Latin America and United States of America [1, 5, 16, 27–29] and higher than that in Nigeria [30]. The higher prevalence in Kenya can be attributed to the increased funding of policies and interventions being implemented by the Kenyan government compared to Uganda [1, 16]. Compared to Uganda, Rwanda’s small population size and the large population density might have contributed to the government’s faster success in implementation of family planning programs [1, 31]. Furthermore, use of government supported community health workers in providing short term methods in the community and the widely utilized community based health insurance program have increased accessibility to modern contraceptives [1, 16, 32, 33]. Although most young women in Kenya and Rwanda get their modern contraceptives from the free public sector facilities, most of the young women in Uganda access contraceptives from private sources with out-of-pocket expenditures [1, 2, 34]. This could as well contribute to the observed lower prevalence rate in Uganda. The modern contraceptive utilization rates in Europe and Latin America are approximately 50% [28] and 30% [5] respectively which are much higher than what we observed in our study. One of the reasons that can explain this is the higher gender equality in Europe which makes women empowered to make decisions regarding contraceptive use [28]. Another explanation could be the higher affluence in Europe compared to Uganda [28]. Adolescents belonging to affluent families could easily access contraceptives through private or public facilities.

The prevalence of contraceptive utilization among the adolescents in our study was lower than that in the older UDHS 2016 participants [22]. Furthermore, Li et al. analyzed 261 DHS and Multiple Cluster Indicator Surveys’ datasets from 103 low- and middle-income countries between 2000 and 2017 showed that adult women aged 20 to 34 years had higher average contraceptive rate of 43.5% which is way higher compared to the finding in our study [35]. The lower utilization rate among adolescents could be attributed partly to the financial constraints of accessing the contraceptives, poor contraceptive knowledge, and limited availability of adolescent friendly health services [35].

Married adolescents were more likely to utilize modern contraceptives compared to unmarried adolescents. Married adolescents are more likely to afford contraceptives compared to their unmarried counterparts due to partner support [36]. Marital status has been shown to be associated with modern contraceptive use in other countries [36–38].Women whose age at first birth was less than 15 years were more likely to utilize modern contraceptives compared to those whose age at first birth was greater than 15 years. This might indicate improved health seeking behavioural which unfortunately comes in later after they have had a birth at a younger age [19]. Furthermore, adolescents that give birth at a younger age are more likely to utilize health facilities for antenatal care, delivery and post-natal care services [22] hence increased exposure to family planning counselling. Age at first birth has been shown to be associated with modern contraceptive utilization in similar studies [19, 39].

Adolescents belonging to the Central region were more likely to utilize modern contraceptives compared to those in the Northern region. The observed regional differences in utilization of modern contraceptives could be attributed to the differences in access to modern contraceptives, sociocultural contexts and job opportunities available to adolescents in the different regions [40]. Most of the young women in Uganda access contraceptives from private sources with out-of-pocket expenditures [1, 2]. Central region unlike the Northern region is the central business region and home to the capital city hence more economically developed with a higher concentration of health facilities, economic opportunities and access to SRH mass communication means such as radios, newspapers [41–43]. All these factors enable easier access and affordability of modern contraceptives and also increase the probability of access to family planning information. Regional differences have similarly been shown to be associated with contraceptive use in various studies [19, 44–46].

Adolescents belonging to the poorest wealth quintile were less likely to utilize modern contraceptive methods compared to those belonging to the middle wealth quintile. Most of the young women in Uganda access contraceptives from private sources with out-of-pocket expenditures [1, 2, 34]. Hence the poor are more likely to have limited access to modern contraceptives due to the out of pocket expenditures to purchase the contraceptives or transport expenditures to free public health facilities [19, 47]. The poor are less likely to be well informed about family planning which can be attributed to the low education levels, less likelihood to own radios, television sets, mobile phones or buy newspapers which limits the likelihood of getting family planning information to enable them make informed healthy choices [39, 48–50]. Wealth has been shown to be associated with modern contraceptive use in other studies [19, 49, 51].

### Strengths

We used a nationally representative sample and weighed the data for analysis and therefore our results are generalized to all Ugandan female adolescents aged 15 to 19 years. Standardised procedures are a requirement of DHS surveys in data collection and validated questionnaires are used which ensures the internal and external validity of the results.

### Limitations

The cross-sectional design is limited by lack of temporality hence causality inferences cannot be made. Most data on the predictors were based on self-reporting and could not be verified through records and hence a possibility of information bias. Data on adolescents below 15 years was not available.

## Conclusion

The findings of this study highlight the influence of age at first birth, region, wealth index and marital status as key predictors of contraceptive use among female adolescents in Uganda. The findings further show a need to promote the availability, accessibility and acceptability of modern contraceptives among female adolescents, especially those that reside in the rural areas who are likely to be poorer. Different stakeholders should design targeted and peer mediated interventions due to differences in adolescents according to their wealth index, regions and marital status.

## Supplementary Information

**Additional file 1: Table1. **Independentvariables’ categorization.

## Data Availability

The data set used is openly available upon permission from MEASURE DHS website (URL: https://www.dhsprogram.com/data/available-datasets.cfm).

## Abbreviations

AOR: Adjusted odds ratio
CI: Confidence interval
COR: Crude odds ratio
DHS: Demographic Health Survey
UDHS: Uganda Demographic Health Survey
OR: Odds ratio
SD: Standard deviation
WHO: World Health Organization
SPSS: Statistical Package for Social Science
USAID: United States Agency for International Development
