# Supplementary material for: Prevalence and factors associated with modern contraceptives utilization among female adolescents in Uganda
Source: BMC Womens Health. 2021 Feb 10;21:61. doi: 10.1186/s12905-021-01206-7 (PMC7877106; doi:10.1186/s12905-021-01206-7)
Supplement: Supplementary file 1 — Additional file 1: Table1. Independentvariables’ categorization. [file 12905_2021_1206_MOESM1_ESM.docx]

**Prevalence and factors associated with modern contraceptives utilization among female adolescents in Uganda**

Quraish Sserwanja^1*^, Milton Musaba^2^, David Mukunya^3,4^

^1^Monitoring and Evaluation Department, Doctors with Africa, Juba, South Sudan

Email: qura661@gmail.com

^2^ Department of Obstetrics and Gynaecology, Busitema University, Tororo, Uganda

Email: miltonmusaba@gmail.com

^3^ Department of Public Health, Busitema University, Tororo, Uganda

Email:zebdaevid@gmail.com

^4^ Sanyu Africa Research Institute, Mbale, Uganda

Email:zebdaevid@gmail.com

**Corresponding author**: Quraish Sserwanja

Doctors with Africa, CUAMM

TM Lion Hotel, Juba, South Sudan

Tel: +256782295939

Email: qura661@gmail.com

**Table 1.** Independent variables’ categorization

| **Variable** | **Description** | **Categorization** |
| --- | --- | --- |
| Age | Age of the adolescent | 15-17  18-19 |
| Wealth Index | Wealth index of the adolescents’ households | Poorest  Poorer  Middle  Richer  Richest |
| Level of Education | Highest level of education attended by the adolescent | No Education  Primary  Secondary  Higher |
| Residence | Place of residence | Rural  Urban |
| Region | Region where the adolescents stay/live | North  East  West  Central |
| Marital status | Current marital status | Married/Cohabiting  Not Married |
| Working status | Current working status | Not working  Working |
| Age at first birth | Age when the adolescents had their first childbirth | Less than 15 years  15-19 years |
| Family planning counselling | Attendance of family planning counselling from a health facility in the last 12 months | No  Yes |
